# Physiological Responses of Cigar Tobacco Crop to Nitrogen Deficiency and Genome-Wide Characterization of the *NtNPF* Family Genes

**DOI:** 10.3390/plants11223064

**Published:** 2022-11-11

**Authors:** Hao Guo, Xuyou He, Hao Zhang, Ronglei Tan, Jinpeng Yang, Fangsen Xu, Sheliang Wang, Chunlei Yang, Guangda Ding

**Affiliations:** 1 Microelement Research Center, Key Laboratory of Arable Land Conservation (Middle and Lower Reaches of Yangtze River), Ministry of Agriculture and Rural Affairs, College of Resources and Environment, Huazhong Agricultural University, Wuhan 430070, China; 2 Tobacco Research Institute of Hubei Province, Wuhan 430030, China

**Keywords:** cigar tobacco, NRT1/PTR family, expression profile, physiological response, nitrate transport, nitrogen deficiency

## Abstract

Tobacco prefers nitrate as a nitrogen (N) source. However, little is known about the molecular components responsible for nitrate uptake and the physiological responses of cigar tobacco to N deficiency. In this study, a total of 117 nitrate transporter 1 (NRT1) and peptide transporter (PTR) family (NPF) genes were comprehensively identified and systematically characterized in the whole tobacco genome. The *NtNPF* members showed significant genetic diversity within and across subfamilies but showed conservation between subfamilies. The *NtNPF* genes are dispersed unevenly across the chromosomes. The phylogenetic analysis revealed that eight subfamilies of *NtNPF* genes are tightly grouped with their orthologues in *Arabidopsis*. The promoter regions of the *NtNPF* genes had extensive *cis*-regulatory elements. Twelve core *NtNPF* genes, which were strongly induced by N limitation, were identified based on the RNA-seq data. Furthermore, N deprivation severely impaired plant growth of two cigar tobaccos, and CX26 may be more sensitive to N deficiency than CX14. Moreover, 12 hub genes respond differently to N deficiency between the two cultivars, indicating the vital roles in regulating N uptake and transport in cigar tobacco. The findings here contribute towards a better knowledge of the *NtNPF* genes and lay the foundation for further functional analysis of cigar tobacco.

## 1. Introduction

Nitrogen (N) is one of the most essential elements required by plants [1]. Most plants are unable to directly utilize atmospheric N_2_, which is only accessible to plants that can symbiose with N_2_-fixing soil bacteria [2]. Hence, it is necessary for most plants to take up other N compounds from the surrounding environment for their growth. The two main types of inorganic N that terrestrial plant species mostly take up are NO_3_^−^ and NH_4_^+^. However, they are in short supply in natural as well as agricultural systems [3]. As a result, soil N deficiency is becoming a major concern in agricultural productivity around the world. N deficiency results in a reduction in plant height, leaf chlorosis, and premature senescence of crops, and eventually reduces crop yield [4,5,6]. In fact, agricultural production is strongly reliant on nitrogenous fertilizer, which is now a considerable cost for the effective development of diverse crops across the world [7]. Chemical N fertilizer application has increased crop productivity and contributed to global food security over the past several decades, but it has also posed a serious risk to the environment, including soil acidification, eutrophication, and global warming [8,9]. This is mostly because 50–70% of this expensive input does not get absorbed by plants and instead stays in the soil [10,11]. Understanding the processes of plant response to N shortage is therefore critical for enhancing crop N-use efficiency (NUE) and lowering artificial N fertilization [12].

Plants display complex stress responses at both physiological and morphological levels to regulate their growth and development under N-deficient situations, such as increased N absorption and translocation, remobilization of N from source organs to newly emerging tissues, and changes in carbohydrate partitioning [12,13]. Nitrate (NO_3_^−^) is most prevalent in soils, and its absorption and transport in plants has a considerable impact on NUE [14,15,16]. It is actively absorbed by the roots and leaves, where it is then delivered by several nitrate transporters, each of which has distinct characteristics, throughout the plant [14,15]. The nitrate uptake system is composed of low-affinity transport systems (LATS) and high-affinity transport systems (HATS), which are primarily mediated by nitrate transporter 1/peptide transporter (NRT1/PTR) and nitrate transporter 2 (NRT2), respectively [15,17]. NRT1 belongs to the large PTR family [18]. Research using 31 completely sequenced plant genomes designated these proteins as NPF (NRT1/PTR FAMILY) and classified them into eight separate clades [19].

AtNPF6.3/AtNRT1.1/CHL1, the first functionally characterized NPF protein, was discovered to act as a nitrate transporter in *Arabidopsis thaliana* [20]. Since then, its homologs in numerous plants have been cloned and functionally identified [14,15,16]. There are 53 *NPF* genes in the *Arabidopsis* genome [16]. Thirteen of these were discovered to be related to root-to-shoot transfer, embryogenesis, or N storage [15]. *AtNPF6.3* encodes a nitrate transporter with dual affinity [21]. It also functions as a nitrate sensor that participates in the early response to nitrate signals [22]. *AtNRT1.5*, a bi-directional nitrate transporter, is essential for the influx and efflux of nitrate during root-to-shoot translocation [23]. *AtNRT1.6* is primarily important for delivering nitrate to seeds to facilitate their growth [24], whilst *AtNRT1.8* and *AtNRT1.9* are involved in long-distance transport and the xylem-to-phloem nitrate-loading process [25]. Both *AtNPF2.12* and *AtNPF5.5* are required for adequate nitrate delivery to growing seeds [26]. The functional homologue of *AtNRT1.1* in rice, *OsNRT1.1B/OsNPF6.5*, performs a similar role to *AtNRT1.1*. Both are localized to the plasma membrane, have a nitrate-inducible expression pattern, and are involved in nitrate intake, transport, and signaling [27]. Additionally, it has been demonstrated that *OsNRT1.1B* is involved in controlling the root microbiota to promote the mineralization of organic nitrogen in soil and hence mediate interactions between plants and microbes [28].

Due to their extremely broad substrate specificity, *NPF* transporters often play a vital role in the development and growth of plants [29,30,31]. To date, at least 21 *NPF* members have been shown to have nitrate transport activity in *Arabidopsis* [29]. It is interesting to note that a large number of NPF proteins in plants can transport different substrates, such as abscisic acid [32], gibberellin [33], glucosinolates [34], and peptides [35]. Additionally, a few NPFs have been shown to transport potassium or chloride, for example, *AtNPF7.3/NRT1.5*, which controls pH-dependent K^+^ efflux [36], and *AtNPF2.5*, which possesses chloride efflux activity [37].

There is extensive cultivation and consumption of tobacco worldwide (*Nicotiana tabacum*). Its leaves may be manipulated to create a variety of products which can be smoked or chewed. Furthermore, tobacco is frequently employed as a model plant in molecular and biochemical research, in addition to being an economically valuable crop plant globally [38]. Large amounts of nitrogen fertilizers are used in the agricultural production of tobacco, but more than half of this N is lost into the environment, owing at least partially to poor N uptake and usage by plants [39]. Research shows that the economic value of tobacco leaves can be increased by applying the appropriate amount of N fertilizer [40]. N nutrition also has a significant influence on the concentration and composition of N-containing chemicals in tobacco products, such as nicotine and aromatic heterocyclic molecules [41]. The physical and biochemical properties of various tobacco types vary largely in response to the N nutrient [39,42]. Cigars are a heterogeneous group of tobacco-based combustibles. The main distinction between cigars and cigarettes as a specific tobacco product is that cigars are wrapped in tobacco rather than paper. Cigar tobacco is the most essential raw material used in the production of cigars. When supplied with NO_3_^−^-N instead of NH_4_^+^-N, cigar tobacco is produced more and is of superior quality, demonstrating that nitrate is a preferred N source for the plant’s rapid growth [43]. There have only been a few reports of research demonstrating the sequence identification, regulation, and functioning of the NRT gene in tobacco [44]. However, the potential candidate genes involved in nitrate uptake by cigar tobacco roots are largely unknown.

In this study, we carried out genome-wide investigations of the *NPF* gene family in *N. tabacum* along with several bioinformatic tests of the candidates, including chromosomal location, gene structure and conserved motif, phylogenetic and collinearity relationships, gene duplication, *cis*-element identification, and gene expression profiles, etc. Then, we examined the expression patterns of NPF candidates at various N levels as well as the physiological responses of two cigar tobaccos to N deficit. Our findings pave the way for further study into the molecular and physiological roles of *NPF* family genes in tobacco.

## 2. Results

### 2.1. Genome-Wide Identification and Characterization of the NPF Genes in N. tabacum

**Figure 1:**
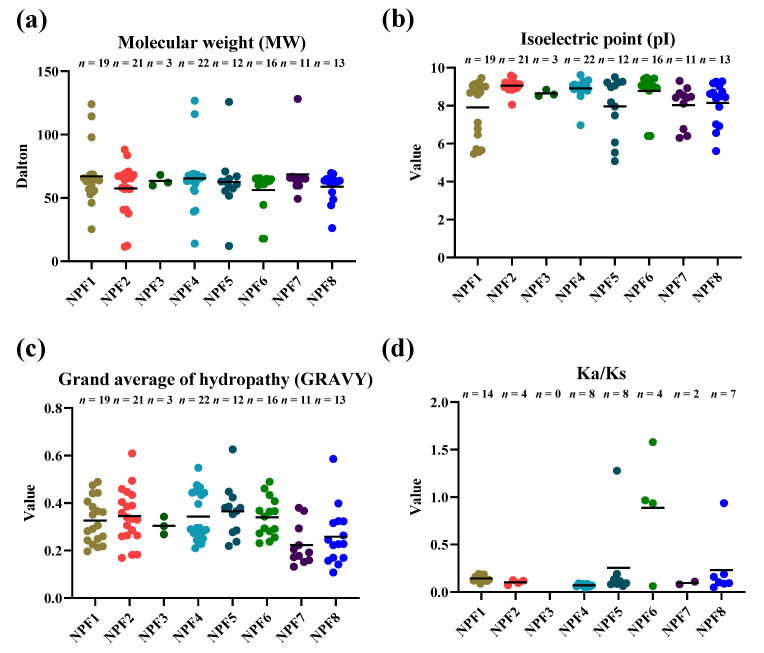
Molecular characterization of the NtNPF proteins in *Nicotiana tabacum*: (**a**) molecular weight; (**b**) theoretical isoelectric point; (**c**) grand average of hydropathy; (**d**) non-synonymous substitution rate (ka)/synonymous substitution rate (ks) ratios.

In *N. tabacum*, 117 *NtNPF* family proteins were discovered using the 53 AtNPF protein sequences from *A. thaliana* as queries (Table S1). Basic information on these *NtNPF* genes, including gene name, genomic position, molecular weight (MW), isoelectronic point (pI), subcellular localization, etc., was analyzed (Figure 1, Table S1). The *NtNPF* genes were named after their corresponding *AtNPF* orthologs. The NtNPF1 proteins have an average of 561 amino acid residues, ranging from 103 (NtNPF2.7g) to 1158 (NtNPF7.3e). The MW was predicted to be between 11.5 (NtNPF2.7g) and 128.2 kDa (NtNPF7.3e) (Figure 1a). In addition, predicted pI values for NPF protein sequences ranged widely from 5.08 (NtNPF5.10c) to 9.61 (NtNPF4.3e), of which 22 were acidic proteins (pI less than seven) and 95 were basic proteins (pI higher than seven) (Figure 1b). The grand average of hydropathicity (GRAVY) varied from 0.11 (NtNPF8.2b) to 0.63 (NtNPF5.10c), indicating that all NtNPF proteins were hydrophilic (Figure 1c). Additionally, it was projected that the majority of NtNPF proteins would be localized in the plasma membrane, indicating that they could be in charge of the trans-membrane trafficking for specialized substates (Table S1).

Furthermore, to assess selection pressure on NtNPFs over evolution, we employed orthologous *NPF* gene pairs from *Arabidopsis* and *N. tabacum* to calculate synonymous (Ks) and non-synonymous (Ka) nucleotide substitution rates, as well as Ka/Ks ratios (Figure 1d, Table S2). Our findings demonstrated that the majority of the Ka/Ks ratios of the orthologous NPF family genes between *Arabidopsis* and *N. tabacum* were less than one, suggesting that the *NtNPFs* may be mostly affected by strong purifying selection [45]. However, two of them were greater than one, suggesting that *NtNPF6.3e* and *NtNPF5.10a* might suffer from positive selection.

### 2.2. Phylogenetic Analysis of the NPFs in N. tabacum

**Figure 2:**
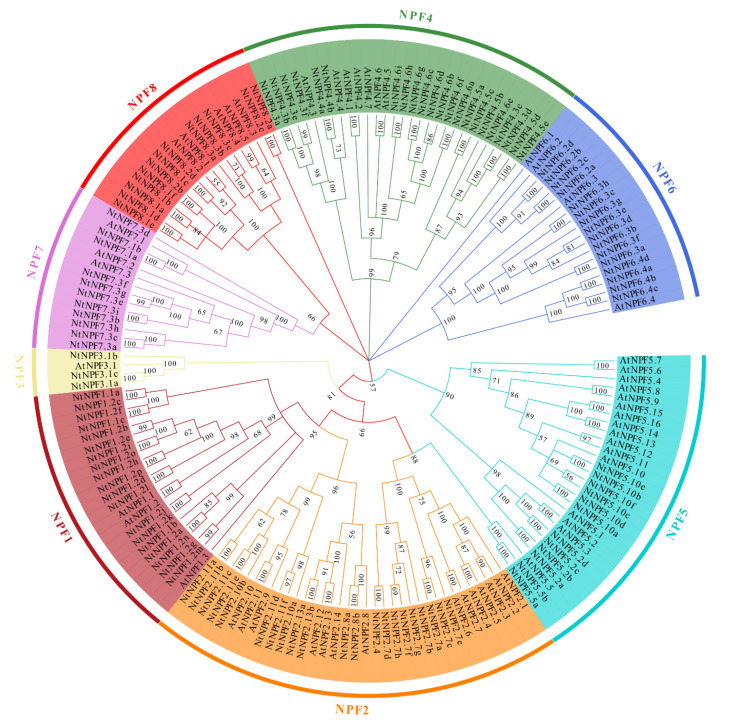
A phylogenetic tree of NPF genes from *Arabidopsis* and *Nicotiana tabacum*. The NPF protein sequences were used to generate the phylogenetic tree in MEGA7 with the neighbor-joining method. The bootstrap value was calculated as the percentage of 1000 trials.

To evaluate the evolutionary relationship between the NPF families of *A. thaliana* and *N. tabacum*, we constructed an unrooted phylogenetic tree including 53 AtNPFs and their orthologs in tobacco using the neighbor-joining (NJ) method with MEGA7.0 software. The results showed that the 117 identified NtNPFs were clustered into eight subfamilies (NPF1-NPF8) according to the percentage of homology between *A. thaliana* and *N. tabacum* NPF proteins (Figure 2). Each NPF subfamily ranged in size from three to 22, with an average of over 14 homologs (Figure 2, Table S1). The largest number of members belongs to the *NtNPF4* subfamily, which is followed by *NtNPF2*, *NtNPF1*, and *NtNPF6*. On the other hand, the *NtNPF3* subfamily has the least amount. The variation in the number of the NPF subfamily might indicate various NPF expansion patterns during the evolution of *N. tabacum*. The phylogenetic tree of *A. thaliana* and *N. tabacum* identified some *NtNPFs* that were closely related to *AtNPFs*, indicating similar physiological functions in nitrate uptake and transport.

### 2.3. Gene Structure and Conserved Motif Analysis of the NPFs in N. tabacum

**Figure 3:**
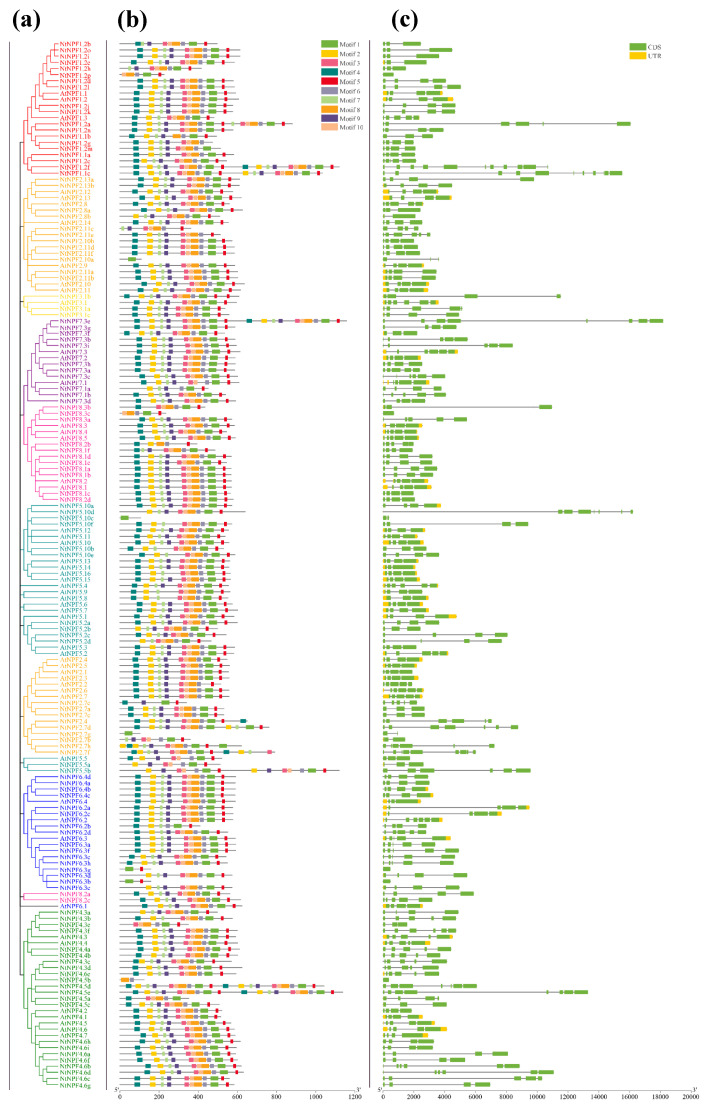
Phylogenetic relationships, architecture of conserved protein motifs, and gene structure of the *NtNPF* genes in *Arabidopsis thaliana* and *Nicotiana tabacum*: (**a**) the *NtNPF* phylogeny; (**b**) the distribution of conserved protein motifs, where each motif is represented by a specific color; (**c**) CDS and UTR which are indicated as green and yellow boxes, respectively. The lengths of each exon and intron can be mapped to the scale given at the bottom.

The examination of the exon–intron structure can provide important insights into gene family evolution. We examined the gene structure of *NPFs* in *N. tabacum* and *Arabidopsis*. A phylogenetic tree was also created to determine whether the exon–intron distribution patterns match the phylogenetic categorization. The results revealed that all *NtNPF* genes had several exons and introns, except five genes which had no intron (*NPF1.2p*, *NPF4.5b*, *NPF6.3g*, *NPF6.3b*, *NPF8.3c*). Most of these genes (59.8%) had untranslated regions (Figure 3c). Furthermore, the *NtNPF* genes in *N. tabacum* have a wide range of intron counts, with the majority having two to five introns. However, the most exons (13) were found in *NPF1.2f*. The number and length of introns and exons were comparable across genes belonging to the same subfamily.

The composition and quantity of the NtNPF conservative motifs were examined using the online MEME program. The 117 NtNPF proteins had a total of 10 conserved motifs (motifs one to 10) (Figure S1). The lengths, conserved sequences, and locations of each motif are given in Table S3. Our findings revealed that the motifs’ lengths ranged from 15 (motifs three and seven) to 41 (motifs one and eight). There were 10 motifs in the majority of the NtNPF proteins. Of them, motif one was prevalent in most of the NtNPFs (Figure 3b). Three NtNPF proteins had only motif one (NPF2.7g, NPF2.10a, and NPF5.10c), but four NtNPF proteins had 20 motifs because of the long protein sequence length (Figure 3b). As predicted, the majority of closely related genes in each subgroup had comparable motif compositions, indicating that these conserved motifs may play important roles in subfamily-specific functions.

### 2.4. Chromosomal Location and Syntenic Analysis of the NPFs in N. tabacum

To better understand how *NtNPF* genes are distributed throughout tobacco chromosomes, chromosomal location analysis was carried out (Figure S2). Forty genes cannot be mapped to a specific chromosome. Of the 24 chromosomes, five (Nt3, Nt11, Nt13, Nt14, and Nt16) had no *NtNPF* gene. The other 77 *NtNPF* genes were widely distributed throughout 19 chromosomes, with a maximum of seven genes on chromosome Nt20. Six *NtNPFs* were discovered on chromosomes Nt4 and Nt18, and five on chromosomes Nt2, Nt8, Nt15, and Nt23, respectively. Chromosomes Nt9 and Nt21 had four copies each, while chromosomes Nt6, Nt10, Nt12, and Nt24 contained three copies each. For the rest of the chromosomes, two *NtNPFs* were found, while chromosome Nt17 only contained one copy.

**Figure 4:**
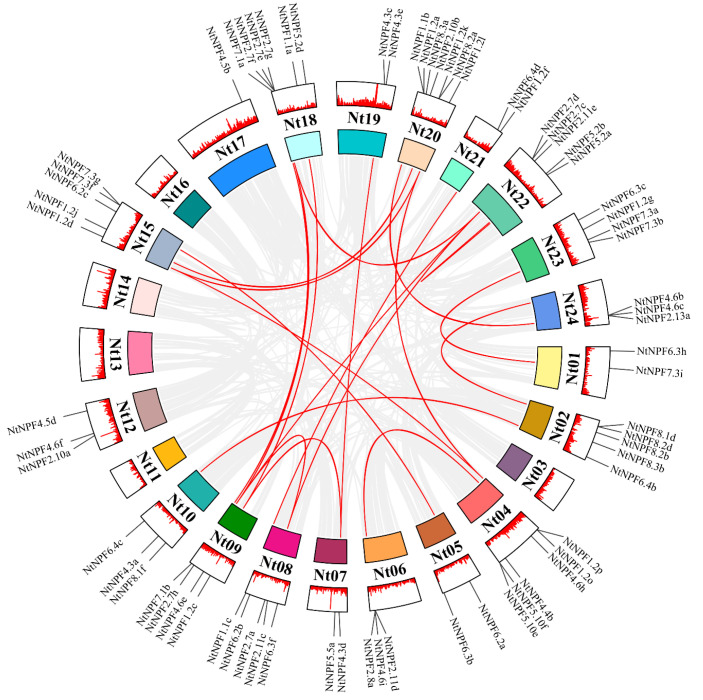
Chromosome distribution and collinearity of the *NtNPF* genes in *Nicotiana tabacum*. The gray lines in the background indicate all the syntenic blocks in the *N. tabacum* genome, and the red lines indicate syntenic *NPF* gene pairs.

Tandem and segmental duplicates play an important role in the expansion of gene families. Tandem duplication occurs mainly in regions of chromosomal recombination, while members of gene families formed by tandem duplication are usually closely aligned on the same chromosome, forming a cluster of genes with similar sequences and functions. Segmental duplication occurs in duplicated genes that are distant or even on distinct chromosomes [46]. Using MCScanX, we examined the gene replication patterns according to the coding sequences in order to uncover the evolutionary history of the NtNPF family (Figure 4). In the Circos plot, 17 gene pairs with segmental duplications were linked by red curves, and one gene pair (*NtNPF5.2a* and *NtNPF5.2b*) was observed on chromosome Nt22. The Ka/Ks ratios between duplicated genes were evaluated to determine duplication events. We discovered that all Ka/Ks ratios in the 18 duplicated events were smaller than 0.40, indicating that the evolutionary process of these genes was mostly influenced by purifying selection pressure (Table S4).

### 2.5. Analysis of Cis-Elements in NPF Promoters in N. tabacum

**Figure 5:**
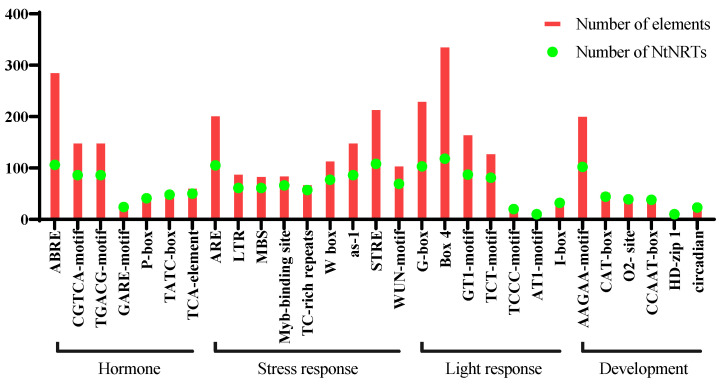
Identification of the *cis*-regulatory elements in the 2 kb promoter regions of the 117 *NtNPF* genes. The *cis*-acting elements were mainly categorized into development, hormone, light response, and stress response-related elements. The bar graph indicates the total number of each *cis*-acting element found in *NtNPF* promoters (red box) as well as the number of *NtNPF* genes which include a specific *cis*-regulatory element (green circle).

Many *cis*-acting elements (CREs) can cooperate with transcription factors to influence gene expression in the upstream promoter regions of genes. To further understand the putative regulatory mechanisms of *NtNPF* genes, the PlantCARE database was utilized to detect over-accumulated elements in the 2.0-kb upstream sequences of the *NtNPF* upstream coding region. Numerous CREs were discovered in addition to the fundamental transcription regulators (TATA box and CAAT box), and they may be categorized into four groups based on their probable functions (Figure 5, Tables S5 and S6). The hormone-related elements mainly include ABRE, CGTCA-motif, TGACG-motif, TCA-element, and ERE (Table S5). Among these, ABRE played a key role in controlling the ABA signaling pathway, which was most prevalent in *NtNPF* promoters. Interestingly, both the CGTCA-motif and TGACG-motif were involved in the MeJA-response regulatory, and the number of these two elements was the same in the *NtNPF* family. There were nine types of stress-responsive elements, which were the most prevalent type in *NtNPF* promoters. For instance, ARE elements were found in 91 (78%) of the *NtNPF* promoters and were crucial for anaerobic induction. Most CREs found in the *NtNPF* genes were related to light response (Figure 5). A total of 289 Box-4 elements were observed in 103 (88%) gene promoters. Other elements, such as G-box, TCT-motif, and GT1-motif, which play an important role in the regulation of light response, were randomly distributed in the promoters of *NtNPFs* (Table S6). The development-related *cis*-elements were relatively fewer than others (Figure 5, Table S6). CAT-box, O2-site, CCAAT-box, and HD-Zip are important during endosperm development, palisade mesophyll cell differentiation, and meristem metabolism (Table S5).

### 2.6. Transcriptional Profiles of the NtNPFs under N Stress and Co-Expression Network Analysis

**Figure 6:**
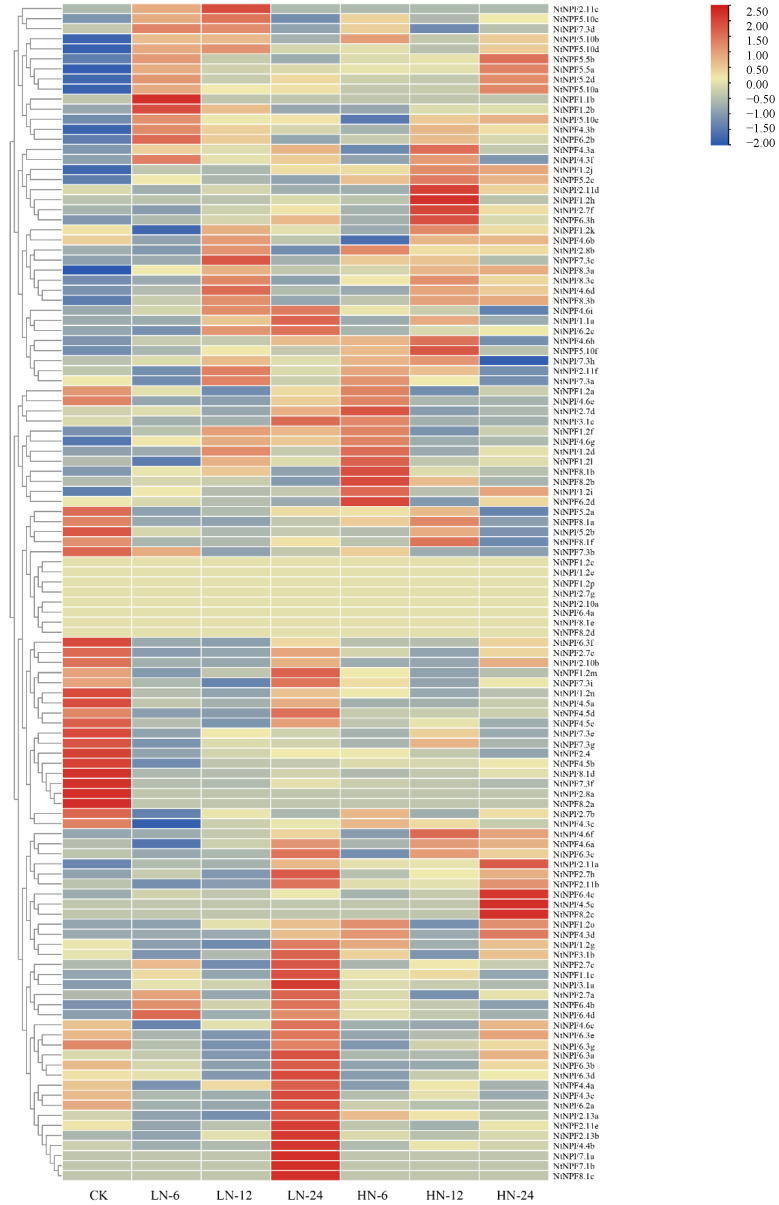
The hierarchical cluster heat map showing *NtNPF* gene expression levels in tobacco root. The seedlings of tobacco were cultivated in a hydroponic system. Plants with approximately 10 cm of leaves in length and with similar sizes were used for nitrate treatments, including high (HN) and low (LN) nitrate concentrations. The root samples were harvested at different time points, including 0 (CK), 6, 12, and 24 h. Three biological replicates for each subgroup were applied.

To examine the expression patterns of *NtNPF* genes, we analyzed the transcription levels of *NtNPF* genes using the RNA-seq data generated from tobacco root treated with a high or low nitrate concentration supplied at different time points (0, 6, 12, and 24 h) (Table S7). The results revealed that the expression patterns of *NtNPFs* changed significantly in response to various N stresses (Figure 6, Table S7), demonstrating their intricate functions in controlling *N. tabacum* development under various N situations. Compared with N-sufficient conditions, 26, 26, and 14 *NtNPFs* were differentially expressed in roots after N starvation for 6, 12, and 24 h, respectively. Under high N conditions, there were 19, 19, and 24 differentially expressed genes (DEGs) at 6, 12, and 24 h, respectively. Approximately 10.3% (12/117), 8.5% (10/117), and 8.5% (10/117) of the DEGs under N limitation were significantly upregulated for 6, 12, and 24 h, and only 12.0% (14/117), 13.7% (16/117), and 3.4% (4/117) of the DEGs were significantly downregulated, respectively. However, there were 9, 11, and 15 genes which were significantly upregulated under high N treatment for 6, 12, and 24 h, respectively.

**Figure 7:**
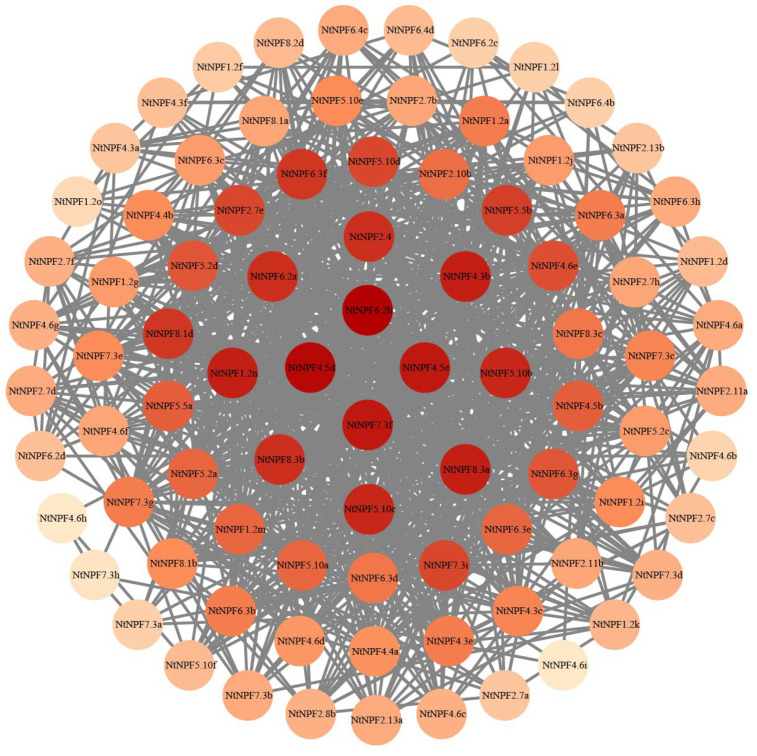
Co-expression networks of *NtNPF* family genes. Cycle nodes represent genes, and the size of the nodes indicates the strength of the node interrelationship by degree value. The intensity of the interactions between two genes is represented by the width of the lines connecting two nodes. The hub NtNPF genes are located in the network’s center.

We then investigated the co-expression interactions between the *NtNPF* genes based on the fragments per kilobase of transcript per million mapped reads (FPKM) values from RNA-seq data to further discover the core *NtNPF* genes under different N treatments (Figure 7). The *NtNPF* family has 870 pairs of co-expression interactions across 117 genes. The hub genes are the top 12 *NtNPF* genes with the highest co-expression interactions with other genes. Among them, *NtNPF6.2b* interacted with 39 *NtNPF* genes, while *NtNPF4.5d* interacted with 38 *NtNPF* genes. Both *NtNPF4.5e* and *NtNPF7.3f* are co-expressed with 36 *NtNPF* genes. With respect to *NtNPF1.2n*, *NtNPF4.3b*, and *NtNPF8.3a*, *NtNPF5.10b* and *NtNPF5.10c*, *NtNPF6.2a*, *NtNPF8.3b*, and *NtNPF2.4*, each pair of these genes had high interaction intensity with 35, 34, and 33 NPF genes, respectively.

### 2.7. 3D Protein Structure, Protein Transmembrane Domain, and Cis-Regulatory Element Analysis of the Hub NtNPFs in N. tabacum

We estimated the transmembrane domains (TMDs) of the 12 major NtNPF proteins using the TMHMM method (Figure S3). Most of the key genes have 8–14 TMDs and are identical to their *Arabidopsis* counterparts. NtNPF4.5e and NtNPF4.5d, however, were predicted to have 19 and 20 TMDs, respectively. As a result, the NPF family may have undergone differentiation and diversity, which may further contribute to functional variations.

Moreover, the three-dimensional (3D) structure of the 12 core NtNPF proteins was predicted by homology modeling using the online program SWISS-MODEL. Ramachandran plots revealed that more than 90% of the residues in all 3D protein models were located in the red core area (the ideal conformation space), demonstrating the models’ high reliability (Figure S5). The beta turn, extended strand, alpha helix, and random coil of all the NtNPFs varied from 45.88 to 52.64%, 10.45 to 16.59%, 2.96 to 4.88%, and 30.98 to 38.22% when matched with the secondary structure prediction (Figure S6, Table S8). The structures of these three proteins (NtNPF1.2n, NtNPF2.4, and NtNPF5.10b) are quite similar, indicating that in addition to sharing a similar protein structure within the same subfamily, they may have had some functional redundancy. Above all, these three-dimensional (3D) protein structures serve as a foundation for investigating their biological roles.

### 2.8. The Physiological Responses of Two Cigar Tobaccos to N Stress

**Figure 8:**
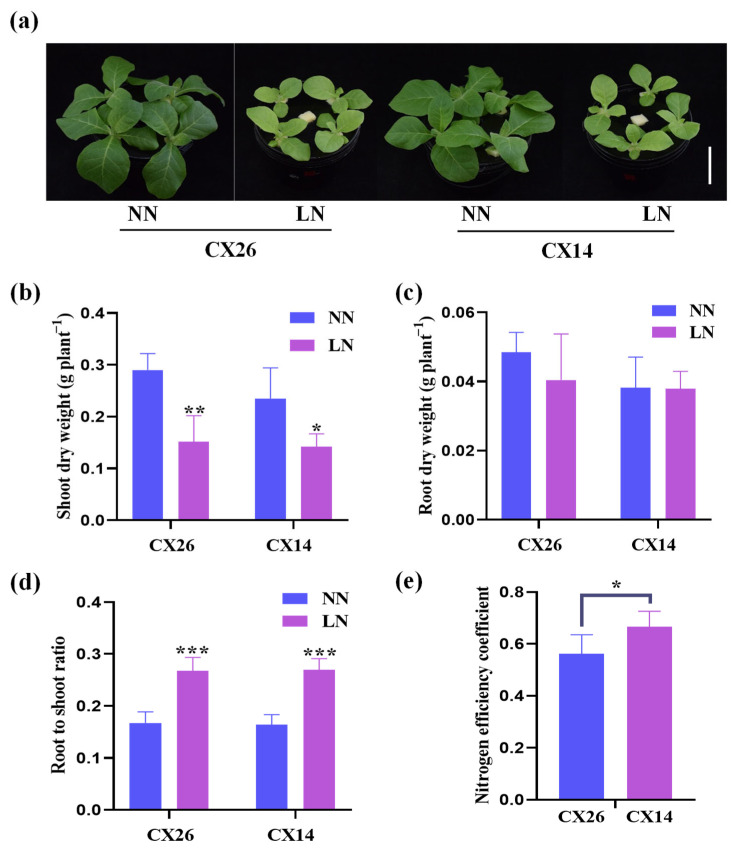
Growth responses of the two cigar tobaccos to two nitrogen levels: (**a**) growth performance of CX14 and CX26 under two treatments; (**b**) shoot dry weight; (**c**) root dry weight; (**d**) root/shoot ratio; (**e**) nitrogen efficiency coefficient. Data represents means ± SD (*n* = 5). A significant difference between CX14 and CX26 under each treatment is indicated by Duncan’s method (*p < 0.05, **p < 0.01, ***p < 0.001).

To investigate the physiological responses of cigar tobacco to N deprivation, two varieties (CX14 and CX26) were used for hydroponic culture to measure the phenotypes under 6 mM (NN) and 0.1 mM (LN) N conditions. Compared with N-sufficient conditions, N stress significantly inhibited the growth of both varieties (Figure 8a). The shoot dry weight of CX26 and CX14 decreased significantly by 47.8% and 39.7%, respectively (Figure 8b). No difference was observed in root dry weight under the LN condition (Figure 8c), but the root/shoot ratio of CX26 and CX14 increased significantly by 60.1% and 64.8%, respectively (Figure 8d). In addition, the N efficiency coefficient is significantly higher in CX14 than in CX26 (Figure 8e). These findings demonstrate that N supply, which is crucial for the growth and development of tobacco, has a more pronounced impact on tobacco shoot than on root, and that CX26 may be more sensitive to N deficiency than CX14.

**Figure 9:**
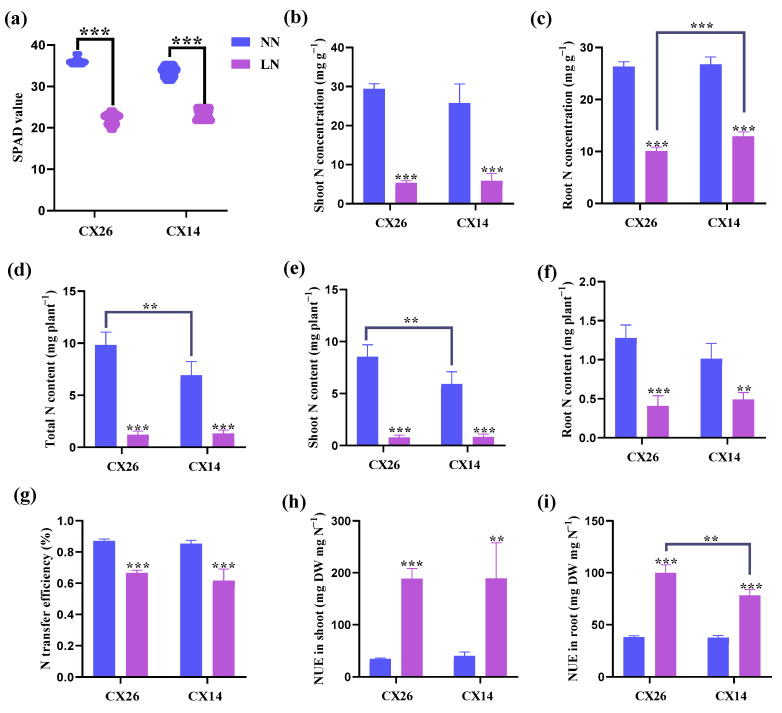
Effects of different nitrogen (N) supplies on N uptake and utilization of CX14 and CX26: (**a**) SPAD value; (**b**) shoot N concentration; (**c**) root N concentration; (**d**) total N content; (**e**) shoot N content; (**f**) root N content; (**g**) N transfer efficiency; (**h**) NUE in shoot; (**i**) NUE in root. Data represents means ± SD (*n* = 5). A significant difference between CX14 and CX26 under each treatment is indicated by Duncan’s method (**p < 0.01, ***p < 0.001).

We then examined the leaf SPAD value, N concentration, N content, and NUE in shoot and root of the two cultivars to further assess the effects of N uptake and utilization. The results showed that the leaf SPAD values of the two cultivars decreased significantly by 38.6% (CX14) and 30.6% (CX26) under N-stress conditions compared with those under N-sufficient conditions (Figure 9a). Under LN treatment, N concentrations in both the shoot and root of the two cultivars are lower than under NN treatment (Figure 9b,c). Notably, CX14 had a higher N concentration in roots under LN conditions compared with CX26 (Figure 9c). However, the shoot N content and total N content of CX26 were 44.8% and 42.1% higher than that of CX14 under NN conditions, respectively (Figure 9d,e). Similarly, the N transfer efficiency of CX26 and CX14 decreased by 23.5% and 27.8% under N-stress conditions compared with that under N-sufficient conditions, respectively (Figure 9g). We then calculated the NUE in the shoot and root of the two varieties. The NUE of both cultivars under the LN condition was significantly higher than that under the NN condition (Figure 9h,i). Moreover, the NUE in the root of CX26 was significantly higher than that of CX14 under N-deprivation conditions, but no difference was observed in the shoot (Figure 9h,i).

### 2.9. The Identification of Key Genes in Nitrate Efficient Uptake and Utilization in Cigar Tobacco

**Figure 10:**
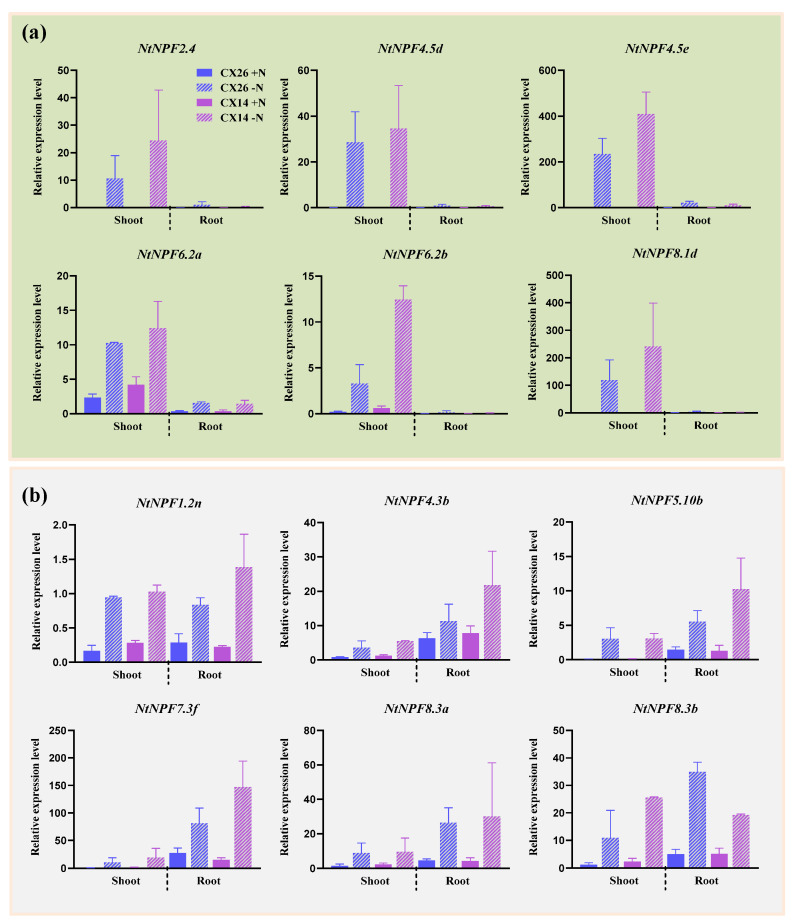
The expression profiles of the 12 *NtNPFs* in different tissues under nitrogen (N) stress by qRT-PCR: (**a**) genes with high expression levels in shoot; (**b**) genes with high expression levels in root. Seedlings at 20 days old were exposed to an N-free nutrient solution for seven days. The roots and shoots were sampled separately for RNA extraction. +N, 6 mM N; −N, 0 mM N.

The shoots and roots of two tobacco seedlings were collected after being cultivated in nutritional solutions with or without N to further investigate the expression patterns of the key *NtNPF* genes during N deprivation. Six *NtNPF* genes (*NtNPF2.4*, *NtNPF4.5d*, *NtNPF4.5e*, *NtNPF6.2a*, *NtNPF6.2b* and *NtNPF8.1d*) were predominantly expressed in the shoot (Figure 10a), whereas the remaining six had high relative expression levels in both cultivars’ roots (Figure 10b). Compared with other genes, *NtNPF4.5e* and *NtNPF7.3f* had the highest expression levels under N deprivation in the shoot and root of both cultivars, respectively. Additionally, significant differences were observed in the expression of the genes from the same subfamily. For instance, *NtNPF4.5d* and *NtNPF4.5e* from the NPF4 subfamily had the highest expression level in the shoot, whereas *NtNPF4.3b* from the same subfamily had a much higher expression level in the root than in the shoot. These findings suggest that members of the same subfamily may function differently.

Interestingly, the expression levels of almost all *NPF* genes induced by N deficiency in CX14 were higher than in CX26. This is consistent with the result that the shoot N concentration and root N concentration of CX14 were higher than those of CX26 under low N conditions (Figure 9b,c). These *NPF* genes might contribute to efficient N uptake and utilization in CX14 under low N conditions. However, *NtNPF8.3b* was the only one whose expression in CX26 was higher than that in CX14 under N-stress conditions in the root, indicating its vital role in N uptake in CX26 under the N-deprivation level. Overall, our findings suggest that multiple *NtNPF* genes may have played important roles in N uptake and utilization in response to N stress.

## 3. Discussion

### 3.1. Composition of the NPF Gene Family in Tobacco

The nitrate transporter 1/peptide family (NPF), nitrate transporter 2 (NRT2), chloride channel (CLC), and SLAC/SLAH (slow anion channel-associated homologues) gene families are the four gene families involved in nitrate transport [15]. Numerous family members with highly conserved NPF domains have been discovered so far in a variety of plant species, such as *Arabidopsis* [18], apple [47], *Brassica* species [48,49], *Camellia sinensis* [50], *Medicago truncatula* [51], poplar [52], rice [19], soybean [53], sugarcane [54], and wheat [55]. The reports show that *NPF* genes serve a variety of vital roles in using N [14,15,16]. The NPF gene family, however, has a complicated composition [19]. A recent comparison of *NPF* members in 36 plant species indicates that distinct NPF subfamilies have considerably varied distributions of NPF members across eudicots and monocots [50]. Compared to monocots, eudicots had a higher proportion of the *NPF1* and *NPF2* subfamilies, whereas monocots had a higher proportion of the *NPF3*, *NPF7*, and *NPF8* subfamilies. By employing genome sequence data from cultivar K326 in the current work, we were able to identify 117 *NPF* genes in *N. tabacum* (Table S1). The *NPF1*, *NPF2,* and *NPF4* subfamilies possess the highest number of members (Table S1, Figure 2). This is different from previous reports on eudicots [19,49,50]. It suggests that these subfamilies may perform additional functions in tobacco and appear to be more active in duplication. The tobacco *NPF* genes are dispersed widely across the chromosomes, with Nt20, Nt4, and Nt18 having the highest gene abundances (Figure S2). Each species has a different number of members of the NPF family. For instance, genes for *Arabidopsis*, *B. napus*, *Medicago truncatula*, rice, tea plant, and *Zea mays* have been reported to be 53, 193, 80, 93, 109, and 79, respectively [50]. The tobacco genome contains a higher number of NPF genes than the majority of other species (Table S1). These findings indicate that the tobacco *NPF* gene family has a complicated composition. The phylogenetic relationships they share with orthologues in other plants are unaffected by this complexity, though. Phylogenetic analysis of the 117 tobacco *NPF* genes revealed that they could be grouped into eight clades, similar to NPF genes from other plant species (Figure 2, Table S1), laying the basis for the proposed systematic nomenclature of tobacco NPF genes [19].

### 3.2. Evolutionary and Structural Characteristics of the Tobacco NPF Family Genes

The NPF family transporters have a high degree of structural conservation and similarity in their sequences [18,19]. Variation in gene structure is commonly thought to be one of the characteristic signs of gene family evolution. We found that *NtNPF* was structurally reasonably conserved within the same subfamily (Figure 3). All 10 motifs are found in the majority of NtNPFs, and no unique motifs from a subfamily have been detected (Figure 3 and Figure S1). Some closely related genes within the same subfamily exhibit the same motif deletion. According to gene structure analysis, most *NtNPF* genes have two to five introns (Figure 3). Similar results have been reported in *Arabidopsis*, *B. napus* [48], soybean [53], and tea plants [50]. In general, gene architecture and motifs were comparable across *NtNPFs* belonging to the same subfamily. The functional diversity of the genes in the tobacco *NPF* family might be attributed to changes in gene and protein structures as well as conserved motifs. Furthermore, the 3D structural model clearly displayed the protein structures of the 12 core NPFs, providing crucial hints for further research into the NPF family’s biological roles in tobacco (Figure 10 and Figure S5).

Higher plants typically have 12 putative TMDs in their NPF transporters [18,47]. We discovered that eight to 20 TMDs are anticipated to be present in the 12 core NtNPF proteins (Figure S4). This is comparable to their *Arabidopsis* counterparts, but the majority of the genes (8/12) are still slightly smaller than in *Arabidopsis*. This is most likely owing to the poor quality of certain assembled genomic sequences, as many possible TMDs failed to meet the cutoff value required for the program to designate them as TMDs [54]. A relaxed selection strength might also lead to fewer TMDs in genes. As a result, it is necessary to determine if the core *NPF* genes in tobacco have the ability to transport nitrate using yeast mutant and transgenic methods in the future. Tandem and segmental duplications are largely responsible for gene family expansion [12]. In this study, 17 gene pairs with segmental duplications and one gene pair (*NtNPF5.2a* and *NtNPF5.2b*) were shown to be associated with segmental duplication and tandem events, respectively (Figure 4, Table S4). These findings suggest that segmental duplication events are mainly responsible for the expanded form of the *NtNPF* gene family. Notably, these genes have only one tandem event, suggesting that the expansion mode may be somewhat conservative under the stress of purifying selection.

### 3.3. NtNPF Genes Function Critically in Nitrogen Uptake and Utilization of Cigar Tobacco

NPF genes have crucial roles and are heavily involved in the complicated processes of N utilization [15,55]. Tobacco *NPF* family members had complex expression patterns at different N levels in their roots, implying that the NPF family has various regulation mechanisms in response to N nutrition (Figure 6). The first cloned nitrate transporter, *AtNPF6.3 (NRT1.1)*, is involved in nitrate absorption in the root, nitrate translocation from root to shoot, and works as a nitrate transceptor to control several physiological, morphological, and molecular responses to nitrate [20,21,22,56,57]. Despite differences in subcellular location, N (nitrate/ammonium) response and usage in two rice orthologues of *NRT1.1*, *OsNPF6.5* and *OsNPF6.3*, can increase rice production and NUE [15,27]. The expression levels of most *NPF6;3/NRT1.1* genes in tobacco roots increased dramatically within 24 h of being exposed to HN and LN conditions (Figure 6), indicating that they could be used as dual-affinity transporters to improve N uptake and utilization [56]. The coordinated expression modification of NPF family members may allow plants to adapt to N fluctuations in the environment. Further verification of the cooperative expression changes of NtNPF family members was provided by the co-expression network analysis, which revealed 870 pairs of co-expression relationships among 117 genes (Figure 7). Similar results were reported in other species [48]. As a result, the NPF family’s cooperative expression may form a large regulatory network, enabling plants to respond quickly to N stress. Importantly, we discovered 12 hub genes from co-expression interactions that may be crucial in the tobacco N regulatory network (Figure 7). Previous studies demonstrated that the *NPF7;3* (*NRT1.5*)-expression level rose dramatically to improve the capacity of N transport from root to shoot, which may determine plant NUE [58]. It is interesting to note that *NPF7;3* has been discovered as the hub gene in the co-expression network of the NPF family in *B. napus* and *tobacco*, indicating that it may have a significant and conservative function in regulating NUE in many species (Figure 7) [48].

To ensure the nutritional quality of tobacco during agricultural production, a substantial amount of N fertilizer is required [39,40,43,59]. As a result, tobacco may be particularly sensitive to changes in N levels in the environment. We found that the shoot growth of both cigar tobacco cultivars was severely suppressed by N stress, but no difference was observed in the root (Figure 8). In addition, the N concentration and content in both tissues of the two cultivars were significantly affected by LN limitation, as well as leaf SPAD values and NUE (Figure 9). The N efficiency coefficient was significantly higher in CX14 than in CX26 (Figure 8e). Moreover, the nutritional quality of cigar tobacco leaves and the concentrations of N and chlorine (Cl) might be affected by N application rate [59]. However, the differences of the two cigar tobacco cultivars in response to normal and N-deficient conditions need to be further investigated at both the maturing and curing stages in the future. Generally, the performance of tobacco is so sensitive to N nutrition that it must be supported by a sophisticated regulatory network. This is further confirmed by the expression levels of 12 hub *NtNPF* genes in the shoot and root of the two cultivars, as they differed largely between these tissues (Figure 10). The expression levels of *NtNPF1;2n*, *NtNPF6;2a* and *NtNPF6;2b* in the shoot increased under LN conditions, suggesting that they may promote N storage in the petiole and subsequent transfer to young leaves [60,61]. The expression of *NtNPF7;3f* is mainly induced by N deficiency in roots, which further promotes N transport from root to shoot, and may affect cigar tobacco NUE [23]. Therefore, *NtNPF* family genes, especially the hub genes, play an important role in regulating N use and response to N deficiency in cigar tobacco. Research showed that the N-efficient tobacco variety was more efficient in N uptake than the N-inefficient variety, especially when grown in LN conditions [42]. We found that the root N concentration of CX14 was significantly higher than that of CX26 under LN conditions, as was the N efficiency coefficient (Figure 8 and Figure 9). This may be attributed to the differentially expressed *NtNPF* genes in response to N stress between the two varieties (Figure 10).

A gene’s expression pattern is typically linked to its CREs [62]. In our study, the promoter regions of *NtNPF* genes possess CREs related to hormones, stress response, light response, and development (Figure 5). These results suggest that, like other species, *NtNPF* transporters might have diverse functions in addition to nitrate and peptide response [15,29]. In apples, *MdABI5* inhibited the expression of *MdNPF7;3* by directly binding to its promoter and thereby impaired ABA-mediated nitrate translocation from root to shoot [63]. Significantly, ABRE is found in the promoter of *NtNPF* genes, showing that the function of NPF genes was conservative throughout the ABA and nitrate interaction process. In addition, the CREs in the promoter region of the *NtNPF* family genes may participate in important life activities, such as anaerobic induction and tobacco C/N metabolism. Overall, *NtNPF* genes play key roles in N uptake and utilization in cigar tobacco and have diverse functions in regulating other metabolic activities. However, it should be noted that smoking is not good for health.

## 4. Materials and Methods

### 4.1. Identification of NPF Family Genes in Tobacco

The *NtNPF* family genes in tobacco were identified using BLASTP (blast protein) and hidden Markov model (HMM) analysis. The protein sequences of AtNPFs were obtained from the *Arabidopsis* Information Resource-10 (http://www.arabidopsis.org/, accessed on 8 April 2022) and used as a query for a BLASTP search against the tobacco genome database (https://solgenomics.net/organism/Nicotiana tabacum/genome, accessed on 09 April 2022) with an e-value of 10^−5^ [64]. The putative NPFs were searched using the local HMMER 3.1 webserver (http://www.hmmer.org/, accessed on 9 April 2022) with default settings. A search from the Pfam database (http://pfam.xfam.org, accessed on 9 April 2022) was performed using the PTR2 domain PF00854. SMART (http://smart.embl-heidelberg.de/, accessed on 9 April 2022) and the conserved domain database (CDD)-Batch search tool (https://www.ncbi.nlm.nih.gov/Structure/bwrpsb/bwrpsb.cgi, accessed on 9 April 2022) were used to further validate the existence of the PTR2 domain in predicted NPFs. The redundant NPFs were removed manually. All NtNPFs sequences, including genes, CDS, proteins, and promoters, were obtained from tobacco genome data files accessible on the Solanaceae Genomics Network (https://solgenomics.net/organism/Nicotiana tabacum/genome, accessed on 9 April 2022). The detected *NtNPF* genes were named following the nomenclature proposed by Léran et al. [19].

### 4.2. Gene Structure, Conserved Motif, and Phylogenetic Analysis

To assess the distribution of exons and introns, the genome of *N. tabacum* was obtained and utilized together with the cDNAs of each gene. Tbtools was used to identify the exon–intron structure and intron phase (0,1,2) [65].

The Multiple Em for Motif Elicitation 5.4.1 (MEME, https://meme-suite.org/meme/, accessed on 1 August 2022) tool was used to examine the conserved motifs of NtNPF encoding proteins with the following parameters: a maximum of 10 motifs, with an optimal width of 6–50 amino acids. The other parameters were left at their default settings. The Interpro database (https://www.ebi.ac.uk/interpro/search/sequence/, accessed on 1 August 2022) was employed to annotate the discovered motifs.

The putative NtNPF sequences in *N. tabacum* and the previously known AtNPF sequences in *A. thaliana* were aligned in ClustalW 1.8.1 using the default alignment settings to create the phylogenetic tree. Additionally, the unrooted phylogenetic tree was constructed using MEGA7 with the neighbor-joining (NJ) method, bootstrapping with 1000 replicates [66]. The final phylogenetic tree was displayed using Evolview V2 (https://evolgenius.info//evolview-v2, accessed on 19 September 2022) [67].

### 4.3. Protein Biophysical Property and Evolutionary Pressure Analysis

The ExPASy-ProtParam tool (http://us.expasy.org/tools/protparam.html, accessed on 7 September 2022) was used to calculate the molecular weight (MW), isoelectric points (IP), and grand average of hydropathy (GRAVY) of NtNPFs. WOLF PSORT, a powerful protein prediction program (https://www.genscript.com/wolf-psort.html, accessed on 7 September 2022), was used to predict subcellular localization. To determine if the NtNPFs encoding sequences are subject to selection pressure throughout evolution, the ratios of synonymous substitution rate (ks) and non-synonymous substitution rate (ka) of homologous gene pairs were analyzed using TBtools [65]. Purifying selection was indicated by a Ka/Ks ratio of less than 1, positive selection by a Ka/Ks ratio of greater than 1, and neutral selection by a Ka/Ks ratio of 0 [68].

### 4.4. Synteny Analysis and Chromosome Localization

The locations of genes and the size of chromosomes were obtained from the gff3 annotation file in order to physically map the NtNPFs on the tobacco genome. Then, the advanced CIRCOS tool in TBtools was used to determine the chromosomal location of NtNPFs [65]. The *N. tabacum* and *A. thaliana* genomes’ collinearity correlations were examined using the multiple collinearity scan toolbox (MCScanX) [69]. Syntenic analysis of NPFs in *N. tabacum* was performed against *A. thaliana* and displayed in TBtools using the dual synteny visualization tool [65].

### 4.5. Cis-Regulatory Elements Analysis

In order to carry out the *cis*-elements analysis, VirtualBox 6.0 was used to retrieve the 2.0 kb upstream promoter sequences of the *NtNPFs* from the tobacco annotation GFF files. The PlantCARE database was then applied to explore the promoter regions of NtNPFs (https://bioinformatics.psb.ugent.be/webtools/plantcare/html/, accessed on 23 May 2022) [70]. The Wordcloud2 package in R was used to display all predicted *cis*-elements except for the TATA-box and CAAT-box.

### 4.6. Heatmap and Co-Expression Network Analysis

The transcriptome data of tobacco root in response to different N treatments was downloaded from the NCBI database with the SRA accession number SRP148925 (https://www.ncbi.nlm.nih.gov/sra/?term=SRP148925, accessed on 27 June 2022) [71]. The FPKM values of NtNPFs, which are converted to log2 fold, were used to generate a heatmap using TBtools [65].

The interaction relationships of gene pairs in the same family were identified by the DeGNServer tool (https://www.zhaolab.org/DeGNServer/index.jsp, accessed on 7 September 2022) based on transcriptome data [72]. Cytoscape was used to display the expected interaction network [73].

### 4.7. Prediction of 3D Protein Structure

The SOPMA online software (https://npsa-prabi.ibcp.fr/cgi-bin/npsa_automat.pl?page=/NPSA/npsa_sopma.html, accessed on 7 September 2022) was used to predict protein structure [74]. Then, the tertiary protein structure was predicted using the Swiss-Model tool (https://swissmodel.expasy.org/, accessed on 7 September 2022) with the homology modeling method [75]. Moreover, the tertiary protein structure was further confirmed using the PROCHECK test in the PDBsum Generate online program (http://www.ebi.ac.uk/thornton-srv/databases/pdbsum/Generate.html, accessed on 7 September 2022), and then visualized by Pymol software [76]. TMHMM 2.0 (http://www.cbs.dtu.dk/services/TMHMM/, accessed on 7 September 2022) was used to examine the key NtNPF genes’ transmembrane domains.

### 4.8. Plant Materials and Stress Treatments

Two cigar tobacco genotypes (CX14 and CX26), which are widely planted in Hubei province and used for cigar production in China, were used for further study here. CX26 is a cigar wrapped tobacco, while CX14 is used to produce cigar filters. Seedlings of the 2 genotypes were cultivated in a growth chamber under the following conditions: 16 h of light and 8 h of darkness, humidity of 60–80%, and light intensity of 300–320 µM proton m^−2^ s^−1^. Before being sown onto seedling trays with a double layer of filter paper, seeds were soaked in deionized water for 2 days in the dark. Then, the uniformed seedlings were grown in a nutrient solution reported previously [77]. For phenotypic experiments, the tobacco seedlings with 6 leaves were treated with 6 mM NO_3_^−^ (NN) or 0.1 mM NO_3_^−^(LN). After 7 days’ growth, the root and shoot of the 2 cigar tobaccos were sampled separately to measure the dry weight as well as the N concentration. For RNA extraction experiments, the plants were kept in an N-free nutrient solution for 7 days. Then, shoots and roots were taken, frozen in liquid N, and kept at −80 °C prior to RNA extraction. The nutrient solution was changed every 3 days. Each treatment included at least 3 biological replicates.

### 4.9. Phenotypic Measurements

The samples were weighed after being dried at 70 °C to a consistent mass, and 0.05 g of each repeat was then digested with H_2_SO_4_-H_2_O_2_. A flow injection analyzer instrument FIAstar 5000 (Foss, Höganäs, Sweden) was used to conduct a quantitative measurement of N. Each sample’s dry weight (DW), N efficiency coefficient (NEC = [DW under LN]/[DW under NN]), N concentration (NC), N content (NA = DW × NC), NUE (DW/NA = 1/NC), and N transfer efficiency (shoot NA/total NA) were computed.

### 4.10. Gene Expression Analysis

Total RNA isolation and cDNA synthesis were carried out as previously reported [68]. The qRT-PCR was performed using the Hieff^®^ qPCR Mix Kit (Yeasen Biotech, Shanghai, China). To standardize transcription levels, *NtL25* was employed as the reference gene [78]. Primer3 (https://bioinfo.ut.ee/primer3-0.4.0/, accessed on 1 September 2022) was used to generate the gene-specific primers utilized in qRT-PCR, which are listed in Table S9. The 2^−ΔΔCT^ approach was used to calculate the relative gene expression level [79]. Three biological replicates were used in the analysis.

### 4.11. Statistical Analysis

SPSS 22 software was used to analyze the data. A Duncan’s multiple range test was employed to compare mean values at the 5% level of significance (*p* < 0.05).

## Data Availability

Not applicable.

## Supplementary Materials

The following supporting information can be downloaded at: https://www.mdpi.com/article/10.3390/plants11223064/s1, Figure S1: The 10 motif sequences of the NPF family protein in *Arabidopsis thaliana* and *Nicotiana tabacum*. Figure S2: Chromosomal distribution of the *NPF* genes in *Nicotiana tabacum*. Figure S3: The presence of *cis*-regulatory elements in the 2.0-kb promoter regions of 12 key NPF genes: (a) over-presentation of the *cis*-regulatory elements in the promoters of 12 genes. The size of the font increases with the number of *cis*-elements, (b) *cis*-elements involved in development, hormones, stress response, and light response are shown in the promoter regions of the *NtNPF* genes. Distinct colors are used to denote different *cis*-elements. Figure S4: Transmembrane domain investigation of *Nicotiana tabacum*’s 12 hub NPF genes. Using the TMHMM tool, the diagram was created based on the complete sequences of the main NtNPF proteins. The transmembrane domains are shown by the purple areas or boxes. Figure S5: Ramachandran maps that are calculated for modeled 3D architectures of the 12 core NtNPF proteins. Areas that are favored, authorized, and typically permitted are indicated by the colors red, brown, and yellow. Figure S6: Predicted 3D structure of the 12 core NtNPF proteins in *Nicotiana tabacum* using the SWISS-MODEL online software. The Pymol software is used to create the structural image. Table S1: Characteristics of the NRT1/PTF family genes in *Nicotiana tabacum* and subcellular localization prediction. Table S2: Non-synonymous (Ka) and synonymous (Ks) nucleotide substitution rates for NPF coding loci in *Arabidopsis* and *Nicotiana tabacum*. Table S3: Predicted values of common motifs identified for individual classes of NtNPF proteins using the MEME suite. Table S4: Gene duplication patterns of the NPF family genes in *Nicotiana tabacum*. Table S5: Summary of selected *cis*-acting regulatory elements found in NtNPF promoters. Table S6: Light-responsive, growth and development-related, hormone-responsive, and stress-responsive *cis*-regulatory elements identified in the promoter region of *NtNPFs*. Table S7: FPKM values of the NtNPFs in different treatments. Table S8: Predicted secondary structure of 12 core *NtNPF* genes in *Nicotiana tabacum*. Table S9: List of q-RT PCR primers used in this study.
