# Supplementary material for: Physiological Responses of Cigar Tobacco Crop to Nitrogen Deficiency and Genome-Wide Characterization of the NtNPF Family Genes
Source: Plants (Basel). 2022 Nov 11;11(22):3064. doi: 10.3390/plants11223064 (PMC9697317; doi:10.3390/plants11223064)
Supplement: Supplementary file 1 [file plants-11-03064-s001.zip › Figures S1-S6.pptx]

## Slide 1
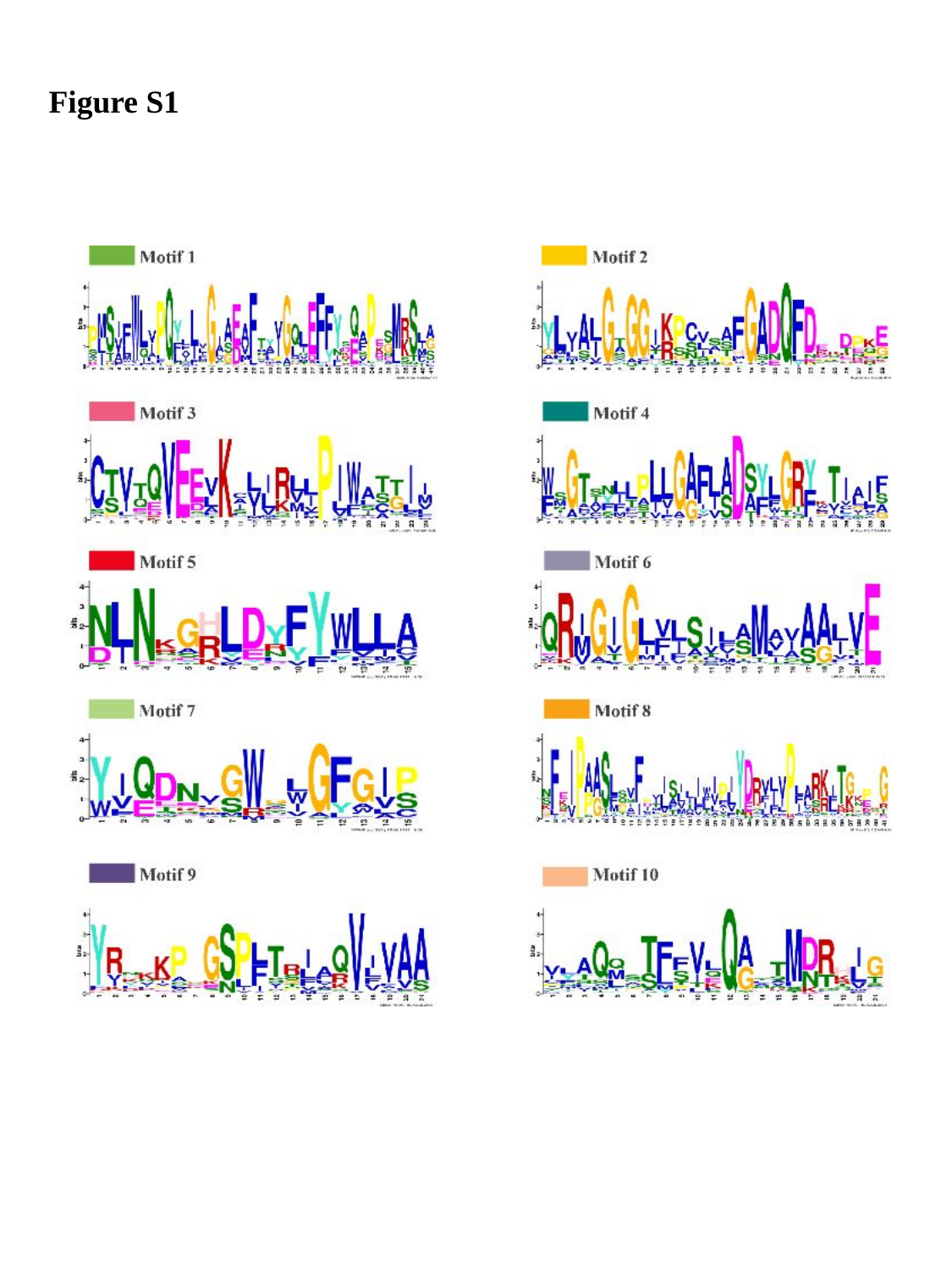

Figure S1

## Slide 2
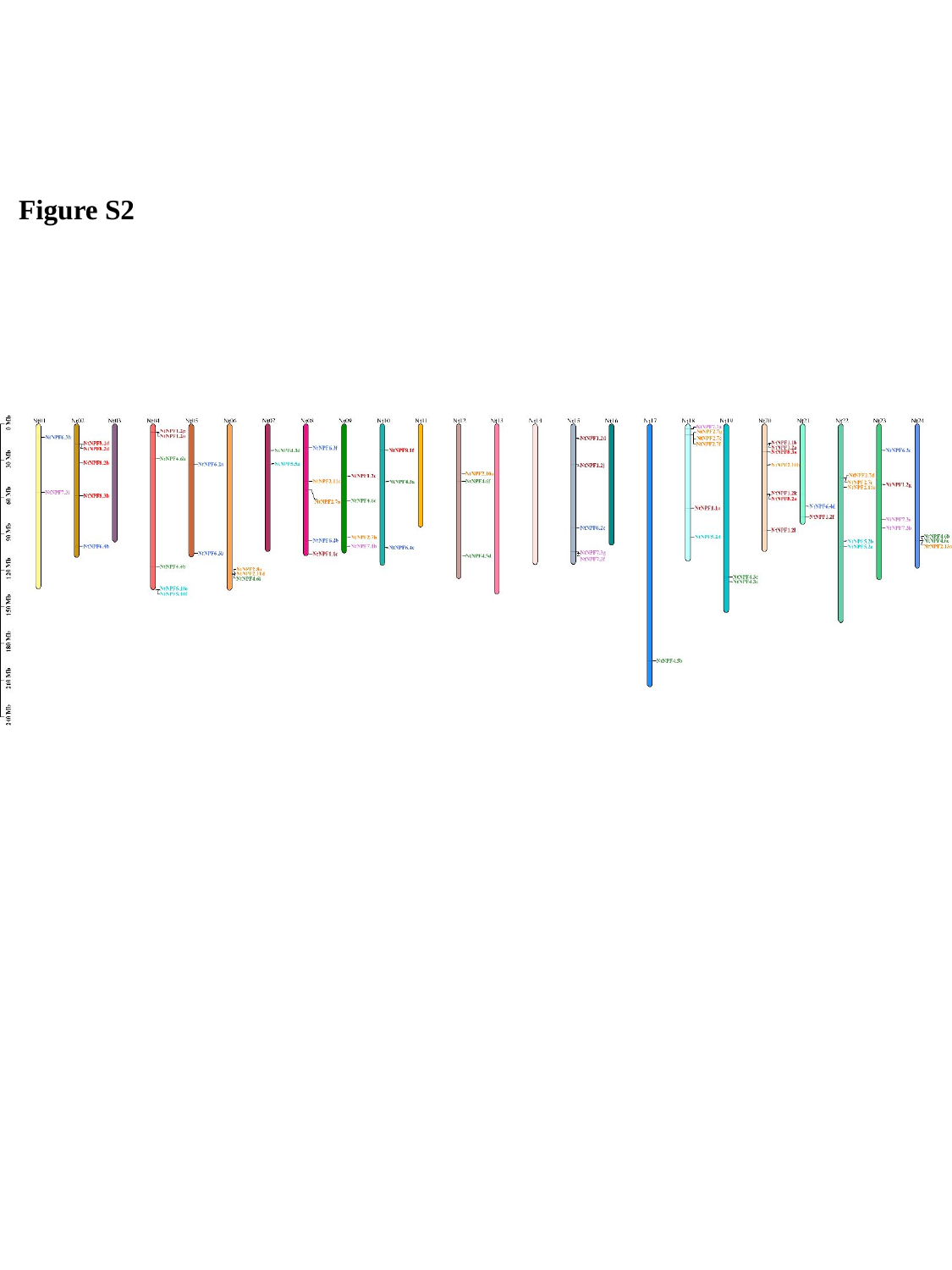

Figure S2

## Slide 3
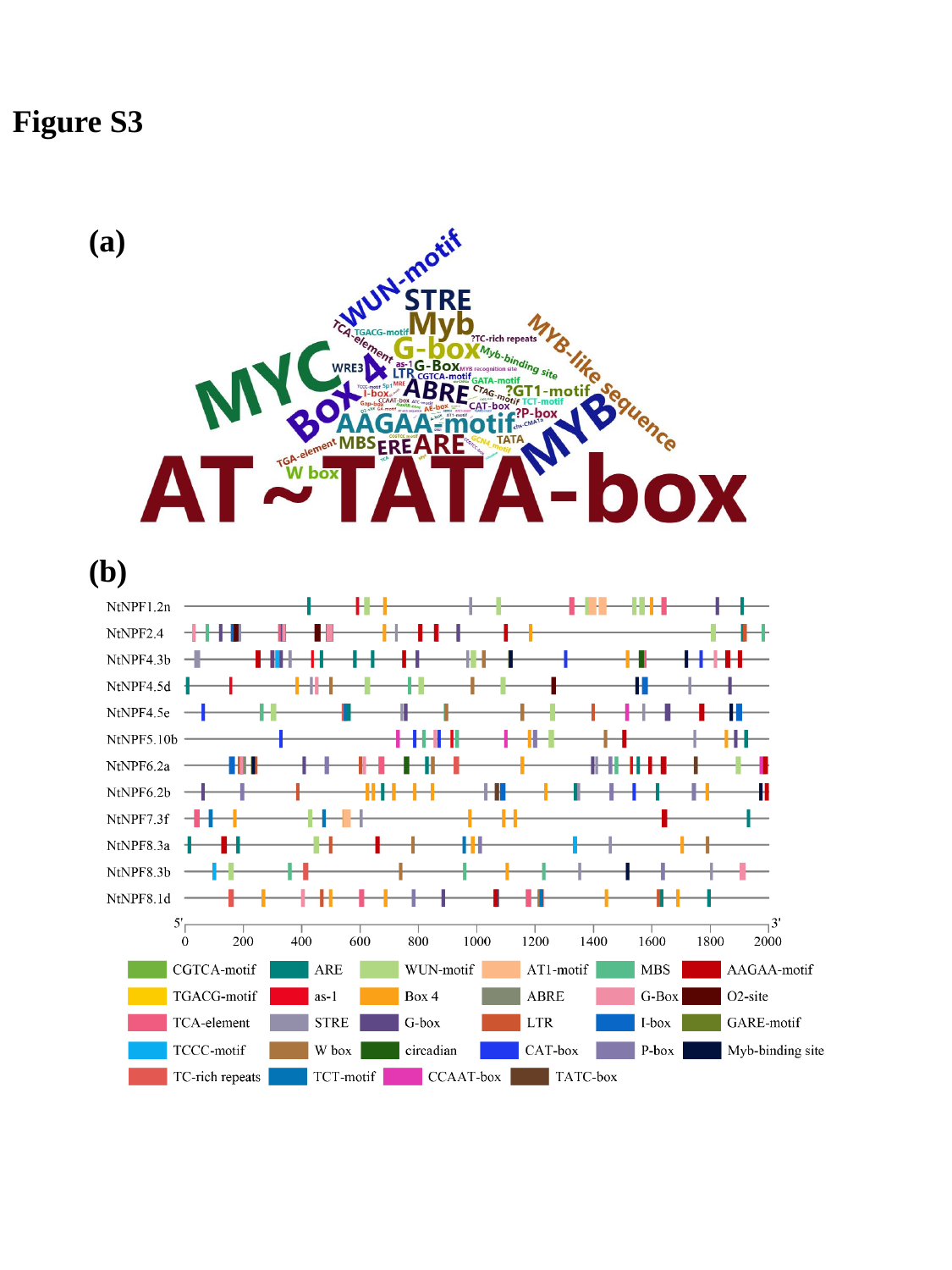

Figure S3
(a)
(b)

## Slide 4
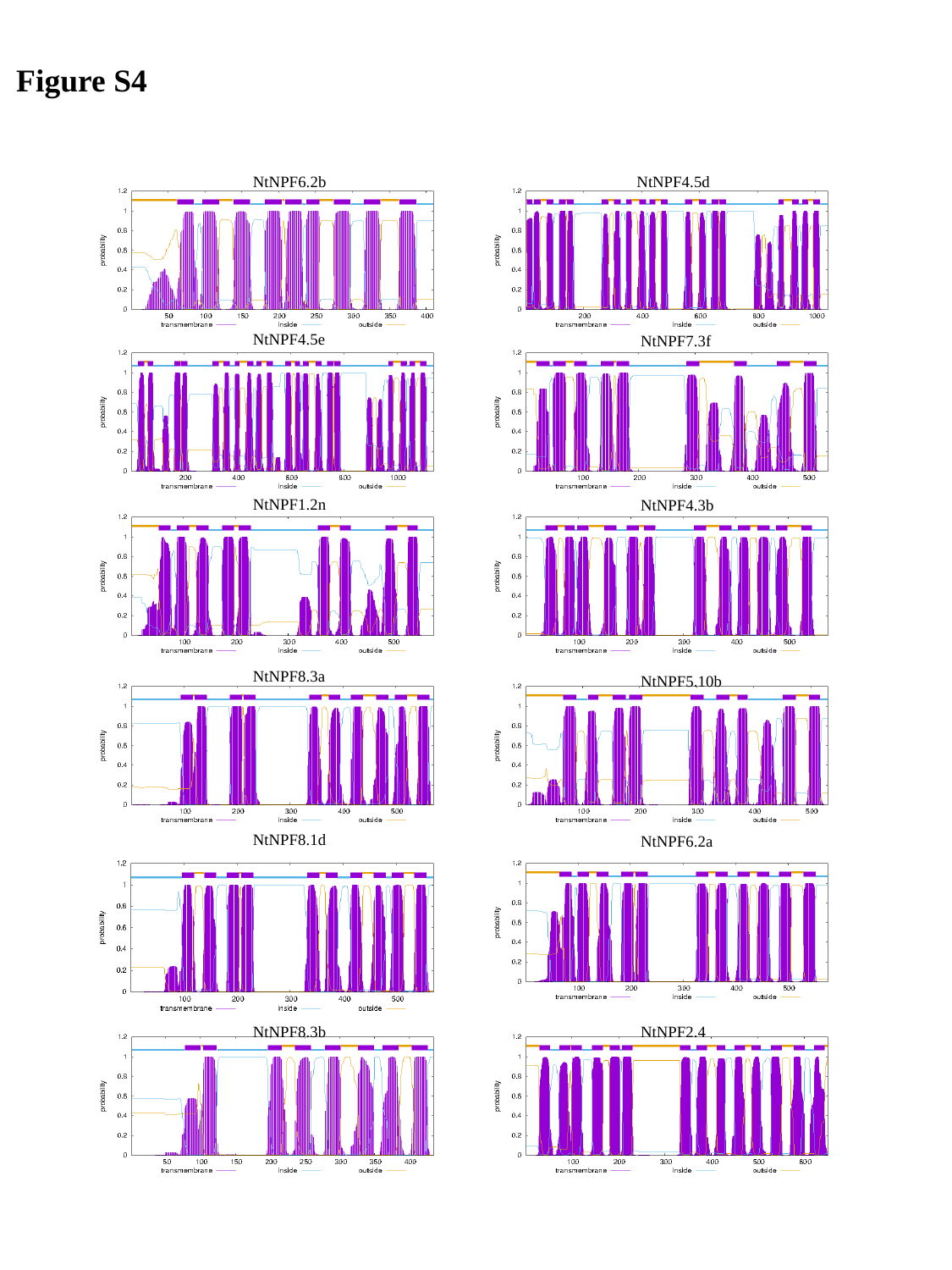

Figure S4
NtNPF4.5d
NtNPF6.2b
NtNPF4.5e
NtNPF7.3f
NtNPF1.2n
NtNPF4.3b
NtNPF8.3a
NtNPF5.10b
NtNPF8.1d
NtNPF6.2a
NtNPF2.4
NtNPF8.3b

## Slide 5
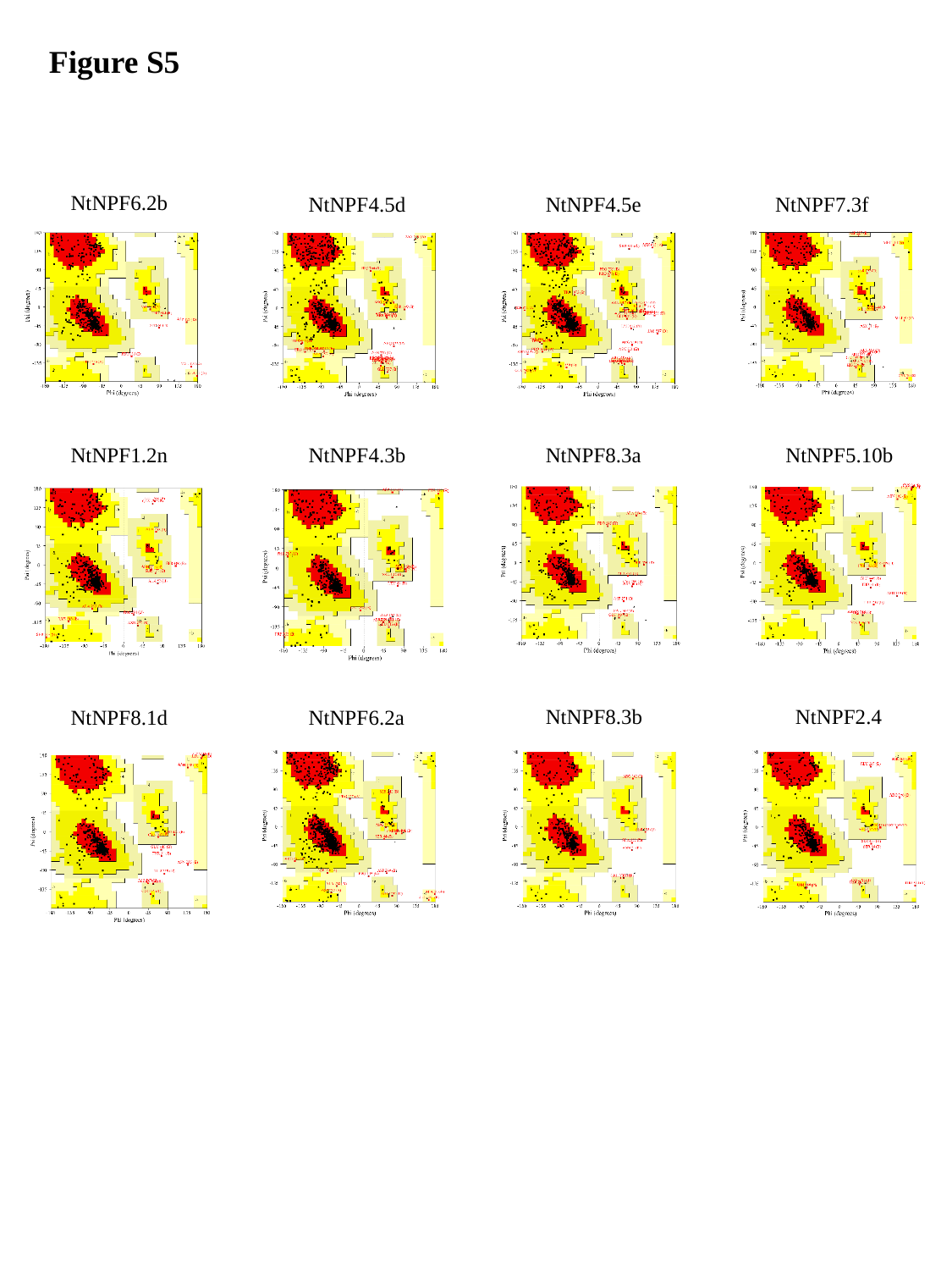

Figure S5
NtNPF6.2b
NtNPF4.5d
NtNPF4.5e
NtNPF7.3f
NtNPF4.3b
NtNPF8.3a
NtNPF5.10b
NtNPF1.2n
NtNPF8.3b
NtNPF2.4
NtNPF8.1d
NtNPF6.2a

## Slide 6
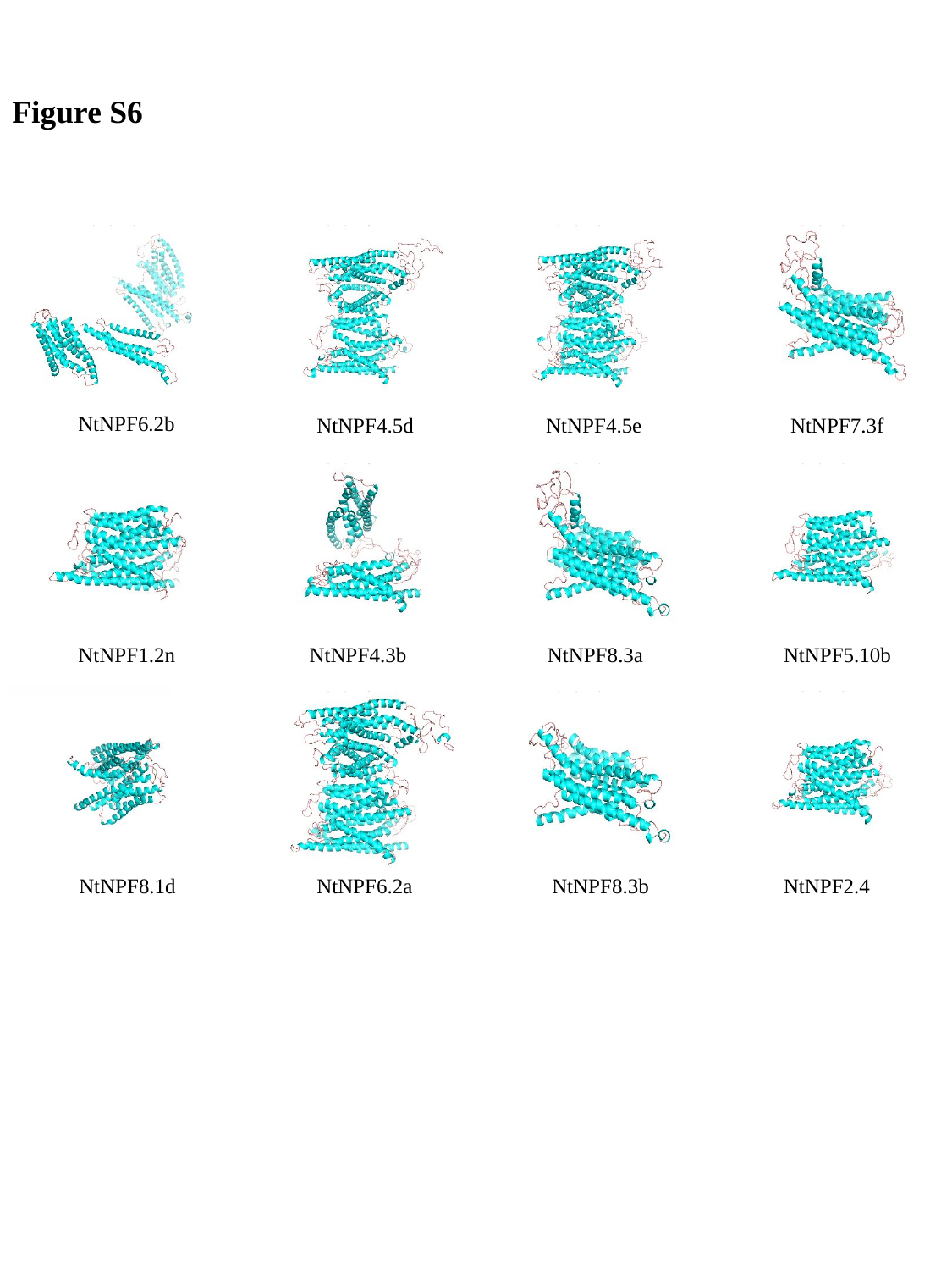

Figure S6
NtNPF6.2b
NtNPF4.5d
NtNPF4.5e
NtNPF7.3f
NtNPF4.3b
NtNPF8.3a
NtNPF5.10b
NtNPF1.2n
NtNPF8.3b
NtNPF2.4
NtNPF8.1d
NtNPF6.2a
